# The Mating-Type Chromosome in the Filamentous Ascomycete *Neurospora tetrasperma* Represents a Model for Early Evolution of Sex Chromosomes

**DOI:** 10.1371/journal.pgen.1000030

**Published:** 2008-03-14

**Authors:** Audrius Menkis, David J. Jacobson, Tim Gustafsson, Hanna Johannesson

**Affiliations:** 1 Department of Forest Mycology and Pathology, Swedish University of Agricultural Sciences, Uppsala, Sweden; 2 Department of Evolutionary Biology, Uppsala University, Uppsala, Sweden; 3 Department of Biological Sciences, Stanford University, Stanford, United States of America; University of Oxford, United Kingdom

## Abstract

We combined gene divergence data, classical genetics, and phylogenetics to study the evolution of the mating-type chromosome in the filamentous ascomycete *Neurospora tetrasperma*. In this species, a large non-recombining region of the mating-type chromosome is associated with a unique fungal life cycle where self-fertility is enforced by maintenance of a constant state of heterokaryosis. Sequence divergence between alleles of 35 genes from the two single mating-type component strains (i.e. the homokaryotic *mat A* or *mat a*-strains), derived from one *N. tetrasperma* heterokaryon (*mat A*+*mat a*), was analyzed. By this approach we were able to identify the boundaries and size of the non-recombining region, and reveal insight into the history of recombination cessation. The non-recombining region covers almost 7 Mbp, over 75% of the chromosome, and we hypothesize that the evolution of the mating-type chromosome in this lineage involved two successive events. The first event was contemporaneous with the split of *N. tetrasperma* from a common ancestor with its outcrossing relative *N. crassa* and suppressed recombination over at least 6.6 Mbp, and the second was confined to a smaller region in which recombination ceased more recently. In spite of the early origin of the first “evolutionary stratum”, genealogies of five genes from strains belonging to an additional *N. tetrasperma* lineage indicate independent initiations of suppressed recombination in different phylogenetic lineages. This study highlights the shared features between the sex chromosomes found in the animal and plant kingdoms and the fungal mating-type chromosome, despite fungi having no separate sexes. As is often found in sex chromosomes of plants and animals, recombination suppression of the mating-type chromosome of *N. tetrasperma* involved more than one evolutionary event, covers the majority of the mating-type chromosome and is flanked by distal regions with obligate crossovers.

## Author Summary

In fungi, mating occurs between individuals of alternative mating-types and there is no dichotomy of individuals into two morphologically different sexes. Nevertheless, in this paper we show that chromosomal regions controlling mating-type identity in fungi share features with the more complex sex chromosomes found in the other eukaryote kingdoms. We have specifically studied the mating-type chromosome in an emerging model-species of filamentous ascomycetes, *Neurospora tetrasperma*, and show that it resembles the sex chromosomes of animals and plants both in failing to recombine with its homologous chromosome over the majority of its length, and having obligate crossovers at the flanking “pseudoautosomal” regions. Furthermore, our data indicate that the evolution of the mating-type chromosome in this species involved more than one successive evolutionary event, each defining an “evolutionary stratum”, a term initially introduced by to represent different sequential steps whereby recombination became arrested between the proto-sex chromosomes in humans. We argue that insight into the evolution of chromosomal sex determination can be gained through the study of alternative, simple, systems, such as *N. tetrasperma*, in which the genomic consequences of reduced recombination *per se* can be disentangled from sex-biased evolutionary forces such as male-biased mutation and dispersal.

## Introduction

Many diverse systems for sex determination have evolved in plants and animals [1]–[3]. One involves physically distinct sex chromosomes, a system thought to have evolved independently many times by suppression of recombination around the sex determination genes, followed by differentiation and degeneration of the non-recombining chromosome [4]. In the fungal kingdom, there is no dichotomy of individuals into sexes bearing different gametes, but instead mating-type identity is determined by inheritance of alleles at mating-type loci. Nevertheless, chromosomal regions controlling mating-type identity in fungi share features with the more complex sex chromosomes of algae, plants and animals [5]. Although mating-type loci consist of one to a few linked genes, and are thus limited to a small genomic region, alleles at the mating-type loci of fungi often differ to the extent that there is no sequence similarity between them [e.g. 6],[7]. Furthermore, complete recombination cessation in the region around the mating-type loci have been reported from several fungal taxa [7]–[9]. However, fungi generally have much smaller regions of suppressed recombination than animal dimorphic chromosomal regions. For example, in *Cryptococcus neoformans* recombination is suppressed on only 6% of a 1.8 Mb chromosome, or ca. 100 kb [8].

The filamentous ascomycete *Neurospora tetrasperma* constitutes an exception in which recombination is blocked over the majority of the chromosome containing the mating-type loci, referred to as the mating-type (*mat*) chromosome. Moreover, the non-recombining region is flanked by distal regions where obligate crossovers are observed [10],[11]. In this species, the large non-recombining region is associated with a uniquely fungal life cycle, called pseudohomothallism, where self-fertility is enforced by maintenance of a constant state of heterokaryosis, normally only observed post-fertilization in this group of fungi. Modified programs of meiosis and sexual spore development lead to the packaging of two haploid nuclei of opposite mating-type (*mat A* and *mat a*) into each *N. tetrasperma* ascospore progeny [12],[13]. The species maintains its ability to outcross by the occasional production of homokaryotic, self-sterile (*mat A* or *mat a*) propagules, both asexual and sexual, which may be isolated to obtain single mating-type component strains. A key feature of meiosis in *N. tetrasperma* is suppressed crossing over on the mating-type bivalent, ensuring that *mat A* and *mat a* will segregate in the first division of meiosis. Although suppressed recombination between *mat* and the centromere would suffice to provide the mechanism for segregation of mating type, the non-recombining region covers a much larger area of the chromosome [10]. The mating-type chromosomes of *N. tetrasperma* therefore resemble the sex chromosomes of animals and plants both in failing to recombine over the majority of their length and having obligate crossovers at the flanking “pseudoautosomal” regions. However, the mechanism initiating the divergence of mating-type chromosomes in *N. tetrasperma* differs from that of animal and plant sex chromosomes, where initiation is suggested to be due to selection for linkage between primary sex-determining alleles and other interacting genes. Such interactions involve alleles with beneficial effects in one sex, but which reduce the fitness of the other sex (e.g. sexually antagonistic genes, [4] and references therein).

Two factors have been suggested to affect recombination between evolving sex chromosomes: the spread of genetic modifiers of recombination rates [14], and chromosomal rearrangements causing chromosome heteromorphism [4]. Both these factors have been suggested to be responsible for the blocked recombination in *N. tetrasperma*. Reciprocal introgression of the mating-type chromosomes between *N. tetrasperma* and its close relative *N. crassa* indicate that both autosomal genes and structural heterozygosity affect recombination in this species [11].

By investigating nucleotide sequence divergence of genes shared between homologous non-recombining chromosomes, insight can be gained into when and how recombination ceased between them, assuming they have been evolving independently since recombination was disrupted. This approach has been used for several systems, including X–Y gametologs of humans [15], mouse [16], dioecious plants [17], W–Z gametologs of chicken [18], and genes located on the mating-type chromosomes of the basidiomycete *Cryptococcus* [19]. All of these systems exhibit “evolutionary strata”, the term initially introduced by Lahn and Page [15] to represent different sequential steps whereby recombination become arrested between the proto-sex chromosomes.

In this study, we compared the level of divergence between alleles on *mat A* and *mat a*-chromosomes from a single wild-type *N. tetrasperma* heterokaryon and found that evolution of the mating-type chromosome in this lineage involved two successive events. The first suppressed recombination over a very large region-at least 6.6 Mbp, or 75% of the chromosome, and was contemporaneous with the split of *N. tetrasperma* from a common ancestor with the outcrossing relative *N. crassa*. The second was confined to a smaller region in which recombination ceased more recently. In spite of the early origin of the first stratum, genealogies of five genes located in this region from strains belonging to an additional *N. tetrasperma* phylogenetic lineage indicate totally independent initiations of recombination suppression in the two lineages. We hypothesize that pseudohomothallism in *N. tetrasperma* evolved in a stepwise manner, and that the steps required to block recombination along the *mat*-chromosome occurred independently in the different lineages in order to facilitate a more efficient first division meiotic segregation of mating type.

## Results

### Allele Divergence of Single Mating-Type Component Strains Originating from the Heterokaryon P581

In order to relate the divergence and evolutionary constraints of alleles within a heterokaryon to the location in the genome, the synonymous (*d*_S_) and non-synonymous to synonymous (*d*_N_/*d*_S_) nucleotide divergence values were estimated for 35 allele pairs of genes of the single mating-type component strains (i.e. homokaryotic *mat A* or *mat a*-strains) originating from the heterokaryotic (*mat A*+*mat a*) strain P581 of *N. tetrasperma* (Table 1). In addition, divergence values (*d*_S_ and *d*_N_/*d*_S_) were estimated between each *N. tetrasperma* allele and the homologous allele of *N. crassa* (http://www.broad.mit.edu/annotation/genome/neurospora/).

**Table 1:** Fungal Material of *N. tetrasperma* and *N. crassa* used in the Study.

Strain^1^		Mating type	
Wild-type strains of *N. tetrasperma*			Geographic origin
Heterokaryon	Homokaryon^2^		
Lineage 1			
P581	FGSC 2508	*A*	Lihue, Hawaii
	FGSC 2509	*a*	Lihue, Hawaii
P556	FGSC 2510	*A*	Hanalei, Hawaii
	FGSC 2511	*a*	Hanalei, Hawaii
P2361	P4371	*A*	Ahipara, New Zealand
	P4372	*a*	Ahipara, New Zealand
Lineage 2			
P505	FGSC2503	*A*	Welsh, Louisiana
	FGSC 2504	*a*	Welsh, Louisiana
P510	FGSC 9051	*A*	Welsh, Louisiana
	FGSC 9052	*a*	Welsh, Louisiana
P4460	FGSC 9030	*A*	Franklin, Louisiana
	FGSC 9031	*a*	Franklin, Louisiana
Manipulated strains^3^			Description
	FGSC3789A	*A*	*N. crassa ro-10 al-2 un-18*
	DJ1544-2a	*a*	Sixth backcross of the *mat a*-chromosome from FGSC 2509 into *N. crassa*

1 FGSC = The Fungal Genetics Stock Center. P = accession number from the Perkins collection of *Neurospora* from nature now curated by FGSC. Lineages are according to the phylogeny of Saenz et al [20].

2 Homokaryons are single mating-type components of the heterokaryons in the left column.

3 See [11] for a description.

**Figure 1:**
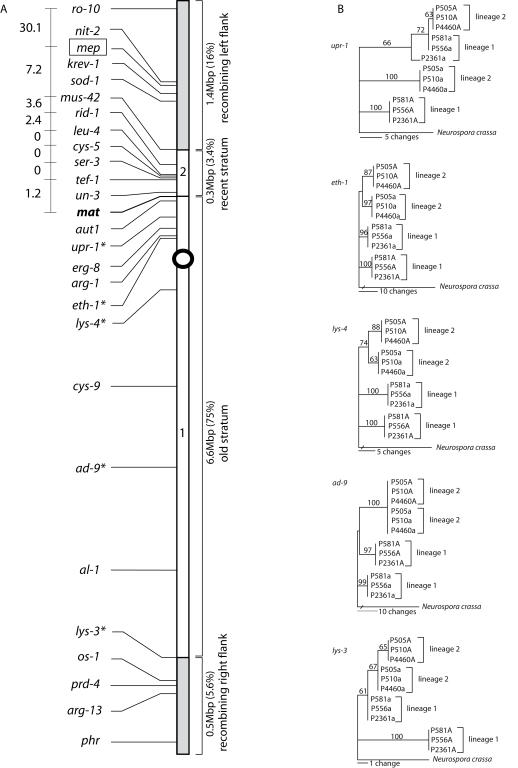
Genetic Map of the *N. tetrasperma* Mating-Type Chromosome Showing Markers used in this Study. A. Left arm of the chromosome is positioned towards the top. Gene order as assumed from the *N. tetrasperma mat a-* chromosome of single mating-type component P581a (2509). Markers with asterisks (*) were also used in the genealogy study; *mep* (boxed) was only used as a phenotype marker in crosses. The two *d*_S_-defined evolutionary strata of strain P581 along the chromosome are indicated and numbered. Numbers to the left of markers *ro-10*–*mat* indicate crossover-frequencies when the *mat a*-chromosome of the *mat a*-component strain of P581 was introgressed into *N. crassa* background and crossed with a *N. crassa mat A* strain. B. One most parsimonious tree of each of the five selected genes of the old stratum. The 12 strains used are single mating-type components of six wild-type *N. tetrasperma* heterokaryons belonging to two different phylogenetic lineages [20]. For clarity, each strain is referred to by the original heterokaryon number followed by the appropriate mating type (*mat A* or *mat a*; Table 1). Bars indicate number of changes, and numbers by branches indicate bootstrap values of above 50% (1000 replicates).

Because of the self-fertilizing nature of the species, genes outside of the regions of blocked recombination are expected to be largely identical between single mating-type component strains isolated from wild heterokaryons. Accordingly, no sequence divergence was found between allele pairs from the single mating-type component strains (i.e. *d*_S_ = 0) of eight genes located at both ends of *mat* chromosome, indicating homogenization of genes in these two distal regions by recombination (Table 2). The region between *mus-42* and *lys-3*, which will hereafter be referred to as the non-recombining part of the *mat* chromosome, in contrast contained 15 divergent allele pairs with *d*_S_-values ranging from 0.013 to 0.082. No divergence was found for two additional genes in this region (*rid* and *cys-5*). The *d*_S_-values of the genes in the non-recombining region, but on either side of *mat*, were found to be significantly different (Mann-Whitney test, p<0.0015); to the right of *mat*, *d*_S_ ranged from 0.047 to 0.082, while *d*_S_ ranged from 0 to 0.04 on the left side of *mat* (Table 2). This difference was significant even when excluding the two non-divergent genes on the left flank (*rid* and *cys-5*; Mann-Whitney test, p<0.0058). Taken together, our data indicate that the evolution of the mating-type chromosome in this lineage involved at least two events, dividing recombination suppression into two strata. The first, larger Stratum 1 includes *mat*, the centromere and the majority of the right arm of the chromosome, and the second, smaller Stratum 2 is restricted to the area left of *mat* (Figure 1A).

**Table 2:** Sequence Divergence between Alleles of Single Mating-Type Component Strains of the Heterokaryotic *N. tetrasperma* Strain P581 (A^T^-a^T^ ) and between each of the Mating-Type Strains and the *N. crassa* Genome Sequence (A^T^-Nc; a^T^-Nc).

Linkage group/region	Locus	Sequence compared (bp)	*d*_S_						*d*_N_/*d*_S_		
			A^T^-a^T^	SE	A^T^-Nc	SE	a^T^-Nc	SE	A^T^-a^T^	A^T^-Nc	a^T^-Nc
LGI											
*Pseudoautosomal*	*ro-10*	603	0	0	0.097	0.026	0.097	0.026	-	0.093	0.093
	*nit-2*	3102	0	0	0.056	0.009	0.056	0.009	-	0.099	0.099
	*krev-1*	551	0	0	0.095	0.028	0.095	0.028	-	0.089	0.089
	*sod-1*	465	0	0	0.071	0.026	0.071	0.026	-	0.041	0.041
*Stratum 2*	*mus-42*	2972	0.013	0.004	0.117	0.014	0.119	0.014	0.105	0.235	0.224
	*rid*	2342	0	0	0.158	0.018	0.158	0.018	-	0.194	0.194
	*leu-4*	1838	0.028	0.008	0.067	0.013	0.057	0.012	0.05	0.054	0.063
	*cys-5*	870	0	0	0.035	0.013	0.035	0.013	-	0	0
	*ser-3*	1084	0.04	0.012	0.082	0.018	0.078	0.017	0.188	0.046	0.049
	*tef-1*	470	0.029	0.017	0.049	0.022	0.059	0.024	0	0	0
	*un-3*	1095	0.04	0.013	0.057	0.015	0.040	0.013	0.06	0.042	0.060
	*mat*										
*Stratum 1*	*aut1*	898	0.055	0.017	0.024	0.011	0.050	0.016	0	0	0
	*upr-1*	4959	0.056	0.007	0.067	0.008	0.071	0.008	0.264	0.349	0.327
	*erg-8*	1410	0.060	0.014	0.077	0.016	0.077	0.016	0.046	0.012	0.023
	*arg-1*	1117	0.047	0.014	0.023	0.010	0.039	0.012	0.025	0	0.031
	*eth-1*	866	0.029	0.012	0.064	0.018	0.054	0.016	0.052	0	0.028
	centromere										
	*lys-4*	1049	0.062	0.016	0.033	0.012	0.045	0.014	0.061	0.040	0.056
	*cys-9*	904	0.082	0.020	0.062	0.017	0.047	0.015	0	0	0
	*ad-9*	621	0.070	0.022	0.092	0.026	0.078	0.024	0.030	0.047	0.027
	*al-1*	1633	0.073	0.014	0.084	0.015	0.092	0.016	0.066	0.081	0.101
	*lys-3*	3348	0.063	0.009	0.067	0.009	0.063	0.009	0.013	0.024	0.013
*Pseudoautosomal*	*os-1*	1683	0	0	0.057	0.012	0.057	0.012	-	0	0
	*prd-4*	1474	0	0	0.146	0.022	0.146	0.022	-	0.031	0.031
	*arg-13*	706	0	0	0.122	0.027	0.122	0.027	-	0	0
	*phr*	1768	0	0	0.102	0.017	0.102	0.017	-	0.091	0.091
LGV											
*Autosomal*	*mus-18*	1738	0	0	0.151	0.020	0.151	0.020	-	0.024	0.024
	*ilv-1*	1738	0	0	0.097	0.016	0.097	0.016	-	0	0
	*cyh-2*	379	0	0	0.032	0.019	0.032	0.019	-	0	0
	*vma-3*	310	0	0	0.012	0.012	0.012	0.012	-	0	0
	*al-3*	1028	0	0	0.066	0.015	0.066	0.015	-	0.153	0.153
	*ro-4*	1862	0	0	0.099	0.021	0.099	0.021	-	0.037	0.037
	*sod-2*	612	0	0	0.043	0.018	0.043	0.018	-	0.248	0.248
	*actin*	1122	0	0	0.011	0.007	0.011	0.007	-	0.106	0.106
	*his-7*	826	0	0	0.110	0.025	0.110	0.025	-	0.015	0.015
LGVI											
*Autosomal*	Bml^*^	251	0		0.055	0.017	0.055	0.017	-	0	0
Linkage group/region	Locus	Sequence	*d*_S_						*d*_N_/*d*_S_		
		compared (bp)	A^T^-a^T^	SE	A^T^-Nc	SE	a^T^-Nc	SE	A^T^-a^T^	A^T^-Nc	a^T^-Nc
LGI											
*Pseudoautosomal*	*ro-10*	603	0	0	0.097	0.026	0.097	0.026	-	0.093	0.093
	*nit-2*	3102	0	0	0.056	0.009	0.056	0.009	-	0.099	0.099
	*krev-1*	551	0	0	0.095	0.028	0.095	0.028	-	0.089	0.089
	*sod-1*	465	0	0	0.071	0.026	0.071	0.026	-	0.041	0.041
*Stratum 2*	*mus-42*	2972	0.013	0.004	0.117	0.014	0.119	0.014	0.105	0.235	0.224
	*rid*	2342	0	0	0.158	0.018	0.158	0.018	-	0.194	0.194
	*leu-4*	1838	0.028	0.008	0.067	0.013	0.057	0.012	0.05	0.054	0.063
	*cys-5*	870	0	0	0.035	0.013	0.035	0.013	-	0	0
	*ser-3*	1084	0.04	0.012	0.082	0.018	0.078	0.017	0.188	0.046	0.049
	*tef-1*	470	0.029	0.017	0.049	0.022	0.059	0.024	0	0	0
	*un-3*	1095	0.04	0.013	0.057	0.015	0.040	0.013	0.06	0.042	0.060
	*mat*										
*Stratum 1*	*aut1*	898	0.055	0.017	0.024	0.011	0.050	0.016	0	0	0
	*upr-1*	4959	0.056	0.007	0.067	0.008	0.071	0.008	0.264	0.349	0.327
	*erg-8*	1410	0.060	0.014	0.077	0.016	0.077	0.016	0.046	0.012	0.023
	*arg-1*	1117	0.047	0.014	0.023	0.010	0.039	0.012	0.025	0	0.031
	*eth-1*	866	0.029	0.012	0.064	0.018	0.054	0.016	0.052	0	0.028
	centromere										
	*lys-4*	1049	0.062	0.016	0.033	0.012	0.045	0.014	0.061	0.040	0.056
	*cys-9*	904	0.082	0.020	0.062	0.017	0.047	0.015	0	0	0
	*ad-9*	621	0.070	0.022	0.092	0.026	0.078	0.024	0.030	0.047	0.027
	*al-1*	1633	0.073	0.014	0.084	0.015	0.092	0.016	0.066	0.081	0.101
	*lys-3*	3348	0.063	0.009	0.067	0.009	0.063	0.009	0.013	0.024	0.013
*Pseudoautosomal*	*os-1*	1683	0	0	0.057	0.012	0.057	0.012	-	0	0
	*prd-4*	1474	0	0	0.146	0.022	0.146	0.022	-	0.031	0.031
	*arg-13*	706	0	0	0.122	0.027	0.122	0.027	-	0	0
	*phr*	1768	0	0	0.102	0.017	0.102	0.017	-	0.091	0.091
LGV											
*Autosomal*	*mus-18*	1738	0	0	0.151	0.020	0.151	0.020	-	0.024	0.024
	*ilv-1*	1738	0	0	0.097	0.016	0.097	0.016	-	0	0
	*cyh-2*	379	0	0	0.032	0.019	0.032	0.019	-	0	0
	*vma-3*	310	0	0	0.012	0.012	0.012	0.012	-	0	0
	*al-3*	1028	0	0	0.066	0.015	0.066	0.015	-	0.153	0.153
	*ro-4*	1862	0	0	0.099	0.021	0.099	0.021	-	0.037	0.037
	*sod-2*	612	0	0	0.043	0.018	0.043	0.018	-	0.248	0.248
	*actin*	1122	0	0	0.011	0.007	0.011	0.007	-	0.106	0.106
	*his-7*	826	0	0	0.110	0.025	0.110	0.025	-	0.015	0.015
LGVI											
*Autosomal*	Bml^*^	251	0		0.055	0.017	0.055	0.017	-	0	0

Each of synonymous nucleotide divergence estimates (*d_S_*) are followed by standard errors (SE). Genes are listed from the left to right flank of the mating-type chromosome according to gene order in *N. crassa*.

The divergence (*d*_S_) between alleles of the *N. tetrasperma* heterokaryon in the first stratum did not differ significantly from the divergence between alleles of *N. tetrasperma* and *N. crassa* (Table 2). Thus, the data suggest that the event creating Stratum 1 was close to the time of the split of *N. tetrasperma* from a common ancestor with *N. crassa.*

The ratio of non-synonymous to synonymous substitutions per site (*d*_N_/*d*_S_) did not differ between alleles of the two *mat* chromosomes and between any of these and *N. crassa*, and no difference in *d*_N_/*d*_S_ was found between *N. tetrasperma* and *N. crassa* when comparing the region of blocked recombination with the other genes of the genome (Table 2).

### Mapping the Boundaries and Estimating the Size of the Non-Recombining Region in Strain P581

To establish the left flank boundary of the non-recombining region, allelic segregation of *mus-42* was scored in 152 heterokaryotic (*mat A* and *mat a*) progeny of the selfed cross of P581. The marker *mus-42* was heteroallelic in all 152 progeny, confirming no crossovers between *mat* and *mus-42* during meiosis. Given a crossover-rate of above 1.95% in this interval in *N. crassa*, we calculate an over 95% probability of detecting a crossover event in 152 offspring (estimated as 1- the probability of finding one crossover in 152 offspring). Thus, *mus-42* is genetically linked to the region of blocked recombination, and our data strongly indicate that the boundary of the non-recombining region is located left of *mus-42*.

The *N. crassa* genome sequence (http://www.broad.mit.edu/annotation/genome/neurospora/) was used to estimate the physical size of the non-recombining region in *N. tetrasperma* strain P581, assuming that the *mat a*-chromosome is collinear with *N. crassa* [11]. The entire region of blocked recombination occupies about 6.9 Mbp (78.4% of the total chromosomal length), but the size of each stratum within the block differs: the older Stratum 1 is 6.6 Mbp (75%), while the more recent Stratum 2 is 0.3 Mbp (3.4%) (Figure 1A).

### Linkage Analysis of Genes on the Mat A-Chromosome of Strain P581

An altered gene order in the *mat a*-chromosome of strain P581 could explain the lack of divergence in *rid* and *cys-5*. A cross between two strains of *N. crassa*, one of which contained an introgressed *mat a*-chromosome originating from P581 (referred to as *mat a* ^T^) was used to infer gene order by crossover frequencies between *mat* chromosome loci (Supporting Information, Table S1). The small number of crossovers among markers in the 83 scored progeny and the lack of double crossovers show tight linkage of the genes, as is known in *N. crassa,* but cannot be used to conclude a definitive gene order. However, all possible orders of these tightly linked genes place them well within the region of blocked recombination.

### Evolutionary Relationship of Five Selected Genes of the Mating-Type Chromosome in Strains Representing Two Lineages of *N. tetrasperma*

The evolutionary history of *mat* chromosome strata may vary among the divergent lineages known within *N. tetrasperma* [20]. To test this possibility, five genes within Stratum 1 were sequenced from single mating-type components of six heterokaryons, representing two phylogenetic lineages of *N. tetrasperma*. The sequences of these genes from the *mat* chromosome were identical for the *mat A*-component strains of each lineage. The *mat a*-component strains of each lineage also had identical gene sequences, except for one intron polymorphism that was found in *upr-1* between *mat a*-component of strain P2361 (FGSC 4372) and the two other *mat a*-component strains of Lineage 1. Synonymous sequence divergence values between allele pairs of the heterokaryons are shown in Table 3. One most parsimonious tree for each of the genes *upr-1*, *eth-1*, *lys-4*, *ad-9* and *lys-3*, and bootstrap support for branches, are shown in Figure 1B.

**Table 3:** Synonymous Sequence Divergence (*d*_S_) between Alleles of Five Genes on the Mating-Type Chromosome in Two Lineages of *N. tetrasperma*.

Locus	Sequence compared (bp)	Strains					
		Lineage 1			Lineage 2		
		P581^1^			P505		
		P556			P510		
		P2361			P4460		
		A^T^-a^T^	A^T^-Nc	a^T^-Nc	A^T^-a^T^	A^T^-Nc	a^T^-Nc
*upr-1*	806	0.061^2^-0.067^3^	0.044	0.044^2^-0.050^3^	0.022	0.056	0.050
*eth-1*	863	0.029	0.059	0.049	0.024	0.049	0.065
*lys-4*	607	0.074	0.029	0.044	0.021	0.021	0.014
*ad-9*	544	0.082	0.108	0.091	0	0.108	0.108
*lys-3*	731	0.039	0.051	0.022	0	0.028	0.028

1 All strains follow Fungal Genetics Stock Center (FGSC) numbers as in Table 1.

2 Strain P2361.

3 Strains P581 and P556. Comparisons between *mat A* and *mat a* component strains originating from six heterokaryotic *N. tetrasperma* strains (A^T^-a^T^), belonging to two phylogenetic lineages [20], and between each of the mating-type strains and the *N. crassa* genome sequence (A^T^-Nc; a^T^-Nc). Genes are listed from left to right on the chromosome based on gene order in *N. crassa.*

Both synonymous divergence data and genealogies confirm that the alleles located on the *mat A* and *mat a*-chromosomes within heterokaryons of all five genes of Lineage 1 diverged early. In contrast, a more recent split of the alleles within heterokaryons are found in Lineage 2 (Table 3; Mann-Whitney test, p<0.0119). Although no synonymous divergence was found for *ad-9* and *lys-3* of Lineage 2 (Table 3), the presence of one non-synonymous change in *lys-3* indicates that recombination is suppressed in this whole region in both lineages (Figure 1B). The genes sequenced here were limited to Stratum 1, and although alleles in this stratum are assumed to have started to diverge in the early evolution of the species (see above), our data imply different evolutionary histories of this part of the *mat* chromosome in the two lineages of *N. tetrasperma*.

### Estimates of Divergence Times

The Kimura 2-parameter genetic distance between *N. crassa* and *N. tetrasperma*, based on intron-data from autosomal genes (i.e. genes located on chromosomes other than the mating-type chromosome; Table 2), was found to be 0.0533. Assuming a divergence time of Eurotiomycetes and Sordariomycetes between 400 to 670 MYA and using the Langley Fitch algorithm to calculate substitution-rate [21], we estimate that *N. tetrasperma* diverged from a common ancestor with *N. crassa* between 3.5 and 5.8 MYA.

## Discussion

Although fungi have no differentiated sexes, i.e. female/male dichotomy of individuals carrying gametes of different sizes, the data presented here confirms that similar mechanisms drive the evolution of sex chromosomes found in the animal and plant kingdoms and the fungal mating-type chromosomes in *Neurospora tetrasperma*. First, the mating-type chromosomes in the pseudohomothallic *N. tetrasperma* fail to recombine over the majority of its length; here we establish that in strain P581 the non-recombining region covers almost 7 Mbp, over 75% of the mating-type chromosome. Previous studies, using the same fungal strain, have shown suppressed recombination of a large portion of the mating-type chromosomes of *N. tetrasperma* [9]–[11]. This study was able to more precisely identify the boundaries and size of the non-recombining region. Notably, the left arm of the non-recombining region is shorter than previously reported [22]; the earlier suggestion that the non-recombining region begins around *nit-2* was not supported here. Instead, the left boundary appears located close to *mus-42* (Figure 1A).

Furthermore, in analogy to systems of sex chromosomes representing all three kingdoms [15]–[19], our data revealed that the evolutionary events leading to the suppression of recombination involved two successive events, resulting in two evolutionary strata, 6.6 Mbp and 0.3 Mbp in size, respectively. Thus, the data suggest that in this fungus stepwise cessation of recombination can take place over a vast genomic region up to 6.6Mbp in size. The event(s) that suppressed recombination are unknown. In the absence of a single, large structural change we may expect a more gradual change in divergence along the chromosome. The simplest possible hypothesis is that Stratum 1 correlates with one large inversion. However, when such a pericentric inversion has been observed on the mating-type chromosome of *N. crassa*, an inversion loop appears to be formed during meiosis, allowing both pairing and crossing over of the inverted region as well as the formation of inviable and unstable progeny [23]. Since such an inversion loop or crossovers do not occur in *N. tetrasperma*, multiple mechanisms for blocking recombination along the mating-type chromosome are likely to be involved. With the upcoming availability of the genome sequence of *N. tetrasperma* (http://www.jgi.doe.gov/) we should be able to disentangle what factor resulted in ceased recombination in this region.

Interestingly, the non-recombining region extends over the majority of the chromosome, although a shorter non-recombining region between *mat* and the centromere would itself be sufficient for the first division meiotic segregation of mating-type that is needed for pseudohomothallism [13]. If one event caused the large Stratum 1, as indicated by our data, it could be the reason for the apparently unnecessary large size. In this context, the reason for the more recent Stratum 2 is obscure. In an earlier study of *C. neoformans* Fraser and co-workers hypothesized that the accumulation of transposable elements would explain the pattern of a gradually growing non-homologous region between the two mating-type chromosomes [19]. Testing the transposon-mediated chromosomal rearrangement hypothesis in *N. tetrasperma* would require further investigation, again possible with the sequenced genome.

A small number of genes showed sequence divergence (*d*_S_) that deviated slightly from the other genes located within the same stratum. For example, in Stratum 2, *rid* and *cys-5* showed no sequence divergence in exons between corresponding alleles (*d*_S_ = 0). In these two genes, no introns are present to support homogenization or divergence between the alleles. However, the mapping data indicate conserved order of nine markers (including *rid* and *cys-5*) located between *ro-10* and *mat* (Supporting Information, Table S1), suggesting that they are not translocated in *N. tetrasperma*. For *eth-1*, located in Stratum 1, we found a *d*s-value of 0.029, which is roughly half the value found for alleles of the other genes in that stratum. As the actual mapping location of *eth-1* was not investigated, the possibility should not be excluded that this gene was recently translocated from the younger evolutionary stratum.

Studies from a diverse range of systems have revealed that lack of recombination *per se* is sufficient for genetic degeneration of a chromosome such as gene loss and null-mutations at protein coding genes, and for transposable element accumulation [4],[24]. The heterokaryotic life-style of *N. tetrasperma*, in which cells during the whole life-cycle carry two nuclei of separate mating-types, would be expected to further favor the erosion of a gene located on these chromosomes, since maintaining function requires an active counterpart on only one of the chromosomes. However, we found no evidence for relaxed selective constraints, as judged from the *d*_N_/*d*_S _comparisons between genes on the mating-type chromosomes and the autosomes, or gene loss in the mating-type chromosomes of *N. tetrasperma*. This observation could be due to the very young age of the system. Alternatively, an occasional homokaryotic part of the life cycle [25], would unmask recessive deleterious mutations and purge these from the population. The accumulation of repetitive elements along the mating-type chromosome remains an interesting target for future research, because these are found to be very early colonizers of non-recombining chromosomes of animal and plant systems [26]–[29].

Multiple phylogenetic lineages exist within *N. tetrasperma*, all of them being pseudohomothallic [20]. The transition from heterothallism to pseudohomothallism in *N. tetrasperma* is associated with loss of mating-type heterokaryon incompatibility. This loss of heterokaryon incompatibility is required to maintain pseudohomothallism and may explain the sexual dysfunction observed when single mating-type strains are outcrossed in the laboratory [30]. The existence of eight-spored outbreeding sister-species to *N. tetrasperma* [i.e. (*N. crassa* (*N. tetrasperma*, PS1, *N.sitophila*)) see [31]; Jeremy Dettman and John Taylor, personal communication] indicate that the non-recombining region formed at or after the split of *N. tetrasperma* from *N. crassa.* We found that Stratum 1 was contemporaneous with the split of *N. tetrasperma* from a common ancestor with *N. crassa*, estimated to be between 3.5 to 5.8 MYA. Assuming that the non-recombining region is a prerequisite for pseudohomothallism would suggest that all lineages of *N. tetrasperma* should share Stratum 1 of the *mat*-chromosome. In contrast, the divergence data and genealogies of five genes located in Stratum 1 suggest that the two different *N. tetrasperma* lineages share a non-recombining region on the mating-type chromosome due to convergent evolutionary events. We hypothesize that pseudohomothallism evolved in a stepwise manner, and that in the early evolution of pseudohomothallism in *N. tetrasperma* there was no recombination block, but that it evolved independently in the different lineages as a selective response for a more efficient pseudohomothallism with absolute first division meiotic segregation of mating type.

Elucidating mechanisms by which sex chromosomes evolve from autosomes has been accelerated by the revolution in genomic science. Considerable insight into plants and animals can be gained through the study of alternative systems, such as *N. tetrasperma*, in which the genomic consequences of reduced recombination *per se* can be disentangled from sex-biased evolutionary forces such as male-biased mutation and dispersal [32],[33]. Thus, the system presented here has the potential to contribute significantly to the general understanding of the forces shaping sex chromosomes, as well as general insights into how levels of polymorphism vary among different regions of the genome.

## Materials and Methods

### Strains and Cultural Conditions

*N. tetrasperma* strains used in this study were obtained from the Fungal Genetics Stock Center (FGSC), Kansas City, KS, or from the Perkins collection at Stanford University, and are listed in Table 1. The Perkins collection is now curated and available from the FGSC. The single mating-type component strains of each heterokaryon (i.e. the homokaryotic *mat A* or *mat a*-strains) were originally obtained through the isolation of homokaryotic sexual or asexual spores occasionally produced by the heterokaryon. The identity of the mating type was confirmed by PCR using allele specific primers [34]. Crosses were made using standard methods on synthetic cross (SC) medium [35] at 25°C. Strains for DNA extraction were grown in minimal medium broth [36] with 1% sucrose for 3 days at 37°C.

### DNA Manipulations

DNA was extracted from fungal vegetative tissue using methods previously described [37]. PCR reactions were performed using the Expand High Fidelity PCR System (Roche Diagnostics, Mannheim, Germany) according to the manufacturer's recommendations, using an Eppendorf epgradient S thermocycler (Eppendorf, Hamburg, Germany). PCR products were purified using ExoSap-IT (Amersham Biosciences, Little Chalfont, UK), and sequencing was performed by Macrogen Inc., Seoul, Korea, utilizing ABI 3730 XL automated sequencers (Applied Biosystems, Foster City, CA). Raw sequence data were analyzed using the SeqMan version 5.01 software from DNASTAR package (DNASTAR, Madison, WI) and BioEdit version 7.0.5.2 [38].

### Evolutionary Divergence of Alleles Located on the Mat Chromosome in a Single *N. tetrasperma* Heterokaryon, P581

Exon sequences from 25 genes located on the mating-type chromosome (also referred to as Linkage Group I: LGI) and ten genes located on autosomes (LGV and LGVI) were chosen for analysis (Table 2). Primers for amplification of nuclear genes were designed from the *N. crassa* genome sequence (http://www.broad.mit.edu/annotation/genome/neurospora/Home.html) by using the PrimerSelect version 5.01 software of the DNASTAR package (DNASTAR, Madison, WI). Primer sequences and information is found in Supporting Information, Table S2. Sequences were PCR-amplified from the separate, homokaryotic, single-mating-type component strains of the wild-type heterokaryon P581: *mat A* (FGSC 2508) and *mat a* (FGSC 2509) (Table 1).

Synonymous and non-synonymous nucleotide divergence values (*d*_S_ and *d*_N_, respectively) were estimated between alleles using DNAsp version 4.10.9 [39]. Comparisons were made between *N. tetrasperma* alleles from the different single-mating-type component strains, as well as between the *N. tetrasperma* alleles and the *N. crassa* genome sequence.

### Mapping the Boundaries of the Non-Recombining Region in Strain P581

To establish the boundary of the non-recombining region on the left flank of LGI of strain P581, recombination was assessed in individual sexual progeny originating from a selfed cross of the heterokaryotic mycelia. Hetero- or homoallelism of *mus-42*, located at the leftmost side of the non-recombining region, was scored in 152 heterokaryotic (*mat A*+*mat a*) progeny, by digesting PCR products obtained by primers TF1 and TR1 (Supporting Information, Table S2) with the restriction enzyme NmuCI (Fermentas Life Sciences, Germany), according to the manufacturer's recommendations. NmuCI has an additional recognition site in the *mus-42* allele from the *mat A*-chromosome of P581, as compared to that of *mat a,* making it possible to separate the two alleles with agarose gel electrophoresis subsequent to amplification and digestion. A recombination event between *mat* and *mus-42* would result in homoallelism of *mus-42* and heteroallelism of *mat* found in a single sexual progeny.

### The Gene Order of the Mat A-Chromosome in Strain P581

Jacobson [11] suggested that the *mat a*-chromosome of *N. tetrasperma* (*mat a* ^T^) is collinear with the *N. crassa mat a* (*mat a* ^C^) chromosome. In order to further establish the location of the genes investigated in this study, we carried out a finer scale linkage analysis of the *mat a* ^T^ chromosome of strain P581 by crossing a fifth backcross progeny of *mat a* ^T^ of P581 introgressed into the *N. crassa* background (DJ1544-2a) [11] and *N. crassa* (FGSC 3789A) (Table 1). First, by DNA sequencing, we confirmed that the parental strain DJ1544-2a contained exclusively *N. tetrasperma* alleles at the genes between *mus-42* and *mat*, allowing for normal linkage testing in this region. Subsequently, the molecular markers *mus-42*, *rid*, *leu-4, cys-5*, *ser-3* and *tef-1*, and the genetic markers *ro-10*, *mep* and *mat*, were scored for 83 progeny from the cross DJ1544-2a×FGSC 3789A. For *mus-42*, *rid*, *leu-4*, *cys-5*, *ser-3* and *tef-1* we scored *N. tetrasperma* and *N. crassa* alleles by PCR-amplification and amplicon digestion using the primer pairs and restriction enzymes TF1 & TR1 (NmuCI), rid-1F2 & rid-1R2 (NmuCI), leu-4F1 & leu-4R1 (EheI), cys-5F & cys-5R (FspBI), ser-3F & ser-3R (HincII) and ef-1aF1 & ef-1aR1 (SmuI), respectively. Primers sequences are found in online Supporting Information, Table S2, enzymes were obtained from Fermentas Life Sciences, Germany, and digestion was performed according to the manufacturer's recommendations. Genetic markers were scored as described previously [11]. Recombination frequencies between the markers were compared to those expected for wild-type *N. crassa*.

### Divergence and Phylogeny of Selected Genes from Multiple Strains of *N. tetrasperma*

The genes *upr-1*, *eth-1*, *lys-4*, *ad-9* and *lys-3* of the homokaryotic, single mating-type components of six *N. tetrasperma* heterokaryotic strains, belonging to either of two well-supported phylogenetic lineages of *N. tetrasperma* (Table 1), were PCR-amplified and sequenced using primers pairs upr-1F1 & upr-1R1, eth-1F1 & ethR1, lys-4F1 & lys-4R1, ad-9F & ad-9R and lys-3F1 & lys-3R1 (Supporting Information, Table S2), respectively. Sequences were aligned for each gene using the Clustal W algorithm of BioEdit version 7.0.5.2 and alignments are available from TreeBASE (study accession no. S1960; matrixes M3612-M3616). Synonymous divergence values (*d*_S_) were estimated between the pairs of alleles of the single mating-type components originating from each of the six heterokaryotic strains of *N. tetrasperma*, as well as between these alleles and the *N. crassa* genome sequence, as described above. Phylogenetic analyses were carried out in PAUP 4.0b [40]. For each gene, we identified maximum parsimony (MP) trees by heuristic searches using the tree bisection-reconnection (TBR) branch-swapping algorithm using *N. crassa* as outgroup. All characters were of equal weight and unordered, and statistical support for phylogenetic grouping was assessed by bootstrap analysis using 1000 replicate datasets with the random addition of sequences during each heuristic search.

## Supporting Information

Table S1 Crossover Frequencies between Mat-Chromosome Loci. Shaded fields show crossover events.
(0.26 MB DOC)

Table S2 Primer Sequences, Annealing Temperatures, and Genomic Locations of Genes According to Gene Order in N. crassa.
(0.23 MB DOC)
