# Supplementary material for: The Mating-Type Chromosome in the Filamentous Ascomycete Neurospora tetrasperma Represents a Model for Early Evolution of Sex Chromosomes
Source: PLoS Genet. 2008 Mar 14;4(3):e1000030. doi: 10.1371/journal.pgen.1000030 (PMC2268244; doi:10.1371/journal.pgen.1000030)
Supplement: Table S1 — Crossover Frequencies between Mat-Chromosome Loci. Shaded fields show crossover events. (0.26 MB DOC) [file pgen.1000030.s001.doc]

Supporting Information, Table S1. Crossover frequencies between mat-chromosome loci. Shaded fields show crossover events.	 
Progeny no. 	Markers on LGI, in gene order based on N. crassa	
	ro-10	mep	mus-42	rid-1	leu-4	cys-5	ser-3	tef-1	mat	
1	T	T	T	T	T	T	T	T	T	
2	T	T	T	T	T	T	T	T	T	
3	T	C	C	C	C	C	C	C	C	
4	T	C	C	C	C	C	C	C	C	
5	T	T	T	T	T	T	T	T	T	
6	T	T	T	T	T	T	T	T	T	
7	T	T	T	T	T	T	T	T	C	
8	T	T	T	T	T	T	T	T	T	
9	T	C	C	C	C	C	C	C	C	
10	T	T	T	T	T	T	T	T	T	
11	T	T	T	T	T	T	T	T	T	
12	T	C	C	C	C	C	C	C	C	
13	T	T	T	T	T	T	T	T	T	
14	T	T	T	T	T	T	T	T	T	
15	T	C	C	C	C	C	C	C	C	
16	T	C	C	C	C	C	C	C	C	
17	T	T	T	C	C	C	C	C	C	
18	T	T	T	T	T	T	T	T	T	
19	T	C	C	C	C	C	C	C	C	
20	T	C	C	C	C	C	C	C	C	
21	T	C	C	C	C	C	C	C	C	
22	T	C	C	C	C	C	C	C	C	
23	T	T	T	T	T	T	T	T	T	
24	T	T	T	T	T	T	T	T	T	
25	T	T	T	T	T	T	T	T	T	
26	T	C	C	C	C	C	C	C	C	
27	T	C	C	C	C	C	C	C	C	
28	T	T	C	C	C	C	C	C	C	
29	T	T	C	C	C	C	C	C	C	
30	T	C	C	C	C	C	C	C	C	
31	T	C	C	C	C	C	C	C	C	
32	T	C	T	T	T	T	T	T	T	
33	T	C	C	C	C	C	C	C	C	
34	T	C	C	C	C	C	C	C	C	
35	T	T	T	T	T	T	T	T	T	
36	T	T	T	T	T	T	T	T	T	
37	T	C	C	C	C	C	C	C	C	
38	T	T	T	T	T	T	T	T	T	
39	T	T	T	T	T	T	T	T	T	
40	T	T	T	T	C	C	C	C	C	
41	C	C	C	C	C	C	C	C	C	
42	C	C	C	C	C	C	C	C	C	
43	C	C	C	C	C	C	C	C	C	
44	C	C	C	C	T	T	T	T	T	
45	C	C	C	T	T	T	T	T	T	
46	C	C	C	C	C	C	C	C	C	
47	C	C	C	C	C	C	C	C	C	
48	C	C	C	C	C	C	C	C	C	
49	C	C	C	C	C	C	C	C	C	
50	C	T	T	T	T	T	T	T	T	
51	C	C	C	C	C	C	C	C	C	
52	C	C	C	C	C	C	C	C	C	
53	C	C	C	C	C	C	C	C	C	
54	C	C	C	C	C	C	C	C	C	
55	C	C	C	C	C	C	C	C	C	
56	C	C	C	C	C	C	C	C	C	
57	C	C	C	C	C	C	C	C	C	
58	C	C	C	C	C	C	C	C	C	
59	C	C	C	C	C	C	C	C	C	
60	C	C	C	C	C	C	C	C	C	
61	C	C	C	C	C	C	C	C	C	
62	C	C	C	C	C	C	C	C	C	
63	C	C	C	C	C	C	C	C	C	
64	C	C	C	C	C	C	C	C	C	
65	C	C	C	C	C	C	C	C	C	
66	C	C	C	C	C	C	C	C	C	
67	C	C	C	C	C	C	C	C	C	
68	C	C	C	C	C	C	C	C	C	
69	C	C	C	C	C	C	C	C	C	
70	C	C	C	C	C	C	C	C	C	
71	C	C	C	C	C	C	C	C	C	
73	C	C	C	C	C	C	C	C	C	
74	C	T	T	T	T	T	T	T	T	
76	C	C	C	C	C	C	C	C	C	
77	C	T	T	C	C	C	C	C	C	
78	C	T	T	T	T	T	T	T	T	
79	C	T	T	T	T	T	T	T	T	
80	C	T	C	C	C	C	C	C	C	
81	C	C	C	C	C	C	C	C	C	
82	T	T	C	C	C	C	C	C	C	
83	C	T	T	T	T	T	T	T	T	
84	C	C	T	T	T	T	T	T	T	
85	C	C	C	C	C	C	C	C	C	
