# Supplementary material for: The Mating-Type Chromosome in the Filamentous Ascomycete Neurospora tetrasperma Represents a Model for Early Evolution of Sex Chromosomes
Source: PLoS Genet. 2008 Mar 14;4(3):e1000030. doi: 10.1371/journal.pgen.1000030 (PMC2268244; doi:10.1371/journal.pgen.1000030)
Supplement: Table S2 — Primer Sequences, Annealing Temperatures, and Genomic Locations of Genes According to Gene Order in N. crassa. (0.23 MB DOC) [file pgen.1000030.s002.doc]

Supporting Information, Table S2. Primer sequences, annealing temperatures, and genomic locations of genes according to gene order in N. crassa. 
Linkage group/ genes	BINCD1 locus	Primers2 	Sequence 5' - 3'	Annealing temp., C0	
LG-I					
ad-9	NCU00843.3	ad-9F	AGTGCCGCATCCTCGTCTT	60.0	
		ad-9R	GGCAATCTTGGCGGTGGCTTCA		
al-1	NCU00552.3	al-1F1 	AAAAATACCAGACTTACAGACAAA	56.7	
		al-1R1	GACAAACTCCAGTAAAAAGAAATA		
		al-1F2	GTGCTGAACGCCGATCTGGTGGTG	62.4	
		al-1R2	CGCCCGCTCCGCATTAAGTGAAAC		
arg-13	NCU02802.3	arg-13F-a	GACTCCGTACCAGCCCAGAC	59.8	
		arg-13R-a	GGAAAATACTTCTTCAACCCATCA		
arg-1	NCU02639.3	arg-1F	AGTCCAAGGGCCGCGTCTGTCT	60.7	
		arg-1R	GGCTTGCCGTCCTTAATCTTCTGA		
aut1	NCU01955.3	aut1F	CTCGACATTCCGCAACACAGG	58.0	
		aut1R	TAAACACCCATGGTAAAGTCGT		
cys-5	NCU02005.3	cys-5F	CATCCATCCATCCATCTTCAACAT	58.8	
		cys-5R	TTTTACCTCCTTTTCTTCTTTCAA		
cys-9	NCU08352.3	cys-9F	CAGGCCGTCACAAAAAGAAAC	59.3	
		cys-9R	CCCTGTACGGCGCTGGTGTC		
tef-1	NCU02003.3	ef-1aF1	TCTCCTGCCACTTTCACATTTCTA	58.5	
		ef-1aR1	CTTGCCGGCCTTGGTCTCCTTCT		
		ef-1aF2	GGTGTCCTCAAGCCCGGTATGGTC	61.3	
		ef-1aR2	GAGGGGAATGGCGATGTGAATGGT		
erg-8	NCU02624.3	erg-8F1	TCGCGATCGCCTCCTTTTTGTTTG	60.3	
		erg-8R1	CGCGACGCTCCTTGATGGTGT		
		erg-8F2	TTCACCGCCTCGCACGCTCTTC	62.8	
		erg-8R2	AACTGGCAACCCTCCCTCCTCTCC		
eth-1	NCU02657.3	eth-1F	GCGAAGCACTACAACGAGGGAACC	61.9	
		eth-1R	CAGCGACACCGATAGCGTAGGAGA		
krev-1	NCU02167.3	krev-1F	ATGATGCGCACTTACGGATTC	56.9	
		krev-1R	GATCGCCCTTCCTCTTTCTCCTC		
leu-4	NCU02010.3	leu-4F1	GTCACAACGTCGGGCACTCTTT	60.1	
		leu-4R1	CCTTCTCGCGCTCGGTAATGTT		
		leu-4F2	CGAGATGGCCGGCACAGAGT	60.9	
		leu-4R2	GCGTTGGCCAAAGATGAAATAGGT		
		leu-4F3	TTACCCTGGCCCTCAACCTCTAC	59.5	
		leu-4R3	AAAGCCAACAAACTCACATCTCGT		
lys-3	NCU03010.3	lys-3F1	CGAGGCACACCCCCAAAGGAC	61.6	
		lys-3R1	GAAGCGCCGCCCACAAGAATC		
		lys-3F2	GGCGCCCAGCTTCTTGTTC	60.1	
		lys-3R2	CCTTGCCGTTGGGGTTCAG		
		lys-3F3	AACCGTTTACCTCAAGAAGT	56.7	
		lys-3R3	AGTGCGGCTGCCCTCCATA		
		lys-3F4	GGGTTACGGCCTCTGGAATGATGA	61.7	
		lys-3R4	GAGCACCACCACGACCACCAGTAG		
		lys-4F1	GGCCCCTACCGTTCTCCA	59.8	
		lys-4R1	CAAAAATGTCGCTGTCGGTGAT		
		lys-4F2	ACGTCAAGAAGGCTGTCAAG	57.5	
		lys-4R2	TCCTCCGCCCGTGCTCATTTTTCT		
mus-42	NCU02053.3	mus-42F1	AACCATGCCTTCAACGAT	55.8	
		mus-42R1	GCCGCTTAGCCAACTT		
		mus-42F2	TGGAAGGCACAGCTCAAGTCTCG	59.6	
		mus-42R2	GAAGCGGATGCCCCAGTTTACC		
		mus-42F3	GCATTGGGCCCGAAAACAGGAGA 	61.5	
		mus-42R3	TTTGCGGGGAGATTGCGGGAGAAG		
		mus-42F4	CCTCGCTTCCTACCGCCGCAACA 	61.5	
		mus-42R4	TCTCCTTCAATCCCCGCTCTTTCA		
		TF1	GCGGCCTGCTGGTTCTCGTC	60.4	
		TR1	CGCCTACTGGGTTGTGCCTTCATT		
nit-2	NCU09068.3	nit-2F1	TCTCGCTCCTCCTTCCAGTTCCTC	61.0	
		nit-2R1	CGTGCTTATGTTTTCGCCGTTGAT		
		nit-2F2	TTCGGAATCAGCCCCTCAAAAATC	61.0	
		nit-2R2	GGTCAATGTGGCCGCTAAACGAGT		
		nit-2F3	CTCGCCAGCAGCCTTATCAGC	59.6	
		nit-2R3	ACCGCGGTTCCGTTTCTTG		
		nit-2F4	AGGGCGCAGCTGGAAACTC	56.1	
		nit-2R4	CATAATAATAAAACATAAAACGAT		
os-1	NCU02815.3	os-1F1 	ATGACTGACGGACCAACTCTC	57.5	
		os-1R1 	ATCTCACCGCGGCATTCAG		
		os-1F2 	CAGGGCATGTGGAACGAACTTACG	59.6	
		os-1R2 	GATGTCGCCGAACGCTCTCACTTG		
		os-1F3 	GTTGACCGACTCTCCATTTTCTGT	57.9	
		os-1R3 	CGGTGCGGCCCTTGTCG		
		os-1F4 	TTGGCGGTACTGGTCTCGGTCTCT	60.4	
		os-1R4 	CTTGCTTTATGGATGTTGGGTTCG		
phr	NCU08626.3	phrF1	GTTTCGCACCATCCGCACCTT 	60.3	
		phrR1	CTTTTCGTCATCTCGCAGTCTT 		
		phrF2	CGGAGTGTTTGGAGATTTACGAA 	59.0	
		phrR2	TCCCCAAGAACAACACTATCCTC 		
prd-4	NCU02814.3	prd-4F1	TGAAGGCAGCAGCACTCT	57.2	
		prd-4R1	GTACCCGTCGACTTCTCCACAC		
		prd-4F2	CATCCGGTGGCTATCTGA	56.6	
		prd-4R2	AAGTCGGGCGAGGTGAGTTC		
		prd-4F3	ACGCCGTTCACCTAGTTCTGGA	57.2	
		prd-4R3	TTTTCTTACCCTTACCCTTACCTT		
rid	NCU11205.3	rid-1F1	GCAGAATGGCCGAGCAAAACC	59.1	
		rid-1R1	CGTGATGGAAGCGTGGATGTAAGA		
		rid-1F2	ACCCGCGGGACAGGAAC 	58.0	
		rid-1R2	TGACGCAAGCCAGACCAGTG 		
		rid-1F3	GTGGTGCTGCCGAGAAT	55.9	
		rid-1R3	GTGCGCACCTTTGGAGA		
ro-10	NCU10696.3	ro-10F	ACAGAACTCGCCCACCAACA	59.9	
		ro-10R	CACTCTATAGCGGCGTCCATCT		
ser-3	NCU02004.3	ser-3F	ATGTCCGACAAAAGCAATACC	59.2	
		ser-3R	TTACTGGATGAGCTGCGAAATCTC		
sod-1	NCU02133.3	sod-1F	GATACCCAAACAAACCGCTTCT	57.8	
		sod-1R	AGTTTTATTTACGCTGCTTGACA		
un-3	NCU01965.3	un-3F1	GTGCCCCTGCCCCGAAGAAGAACG	60.7	
		un-3R1	ATGGGAAGGAGACGGTCAACAAAG		
		un-3F2	CCGCGCTCAGTGGTTTG	58.1	
		un-3R2	AGGCAGTCTTTTGGTATTTGGTG		
		un-3F3	ACCGGCTGGGATATTTTGTTCTT	59.6	
		un-3R3	GCTCCTGTTGCTTCTTGATGGTG		
upr-1	NCU01951.3	upr-1F1	CTCCAGCGGCCAACATCAT	57.2	
		upr-1R1	GAACCCACCCCATCACCTG 		
		upr-1F2-a	GCAGGAAAAAGCGTATGG	55.3	
		upr-1R2-a	GCTGGATCCTGATTATTGGGTTTC		
		upr-1F3	GAAAATACAGGAACAAACTCAA 	55.1	
		upr-1R3	CACACTGCACTTCGTCTTCT 		
		upr-1F4	AAGCAAACAGGATCAGGCACAGTA 	57.5	
		upr-1R4	GCCCAAGGAAGGTCGAGTAGCAGT		
		upr-1F5	TCCTTTGCTTGTTCTTGACTTTC 	58.4	
		upr-1R5	TTCTGCCGCTGCCGTACCCTTTTT		
		upr-1F6	AGACGCTGCTGACGATGCTTCC 	60.8	
		upr-1R6	ATTCCAACGATGCCGCCTTCAG		
LG-V					
actin	NCU04173.3	actinF1	CTTTTCGTCACCACCTAATCTATC	58.1	
		actinR1	GAAGGCGGGGGCGTTGAAAGTCTC		
		actinF2	GAGCACCCCGTCCTTCTTAC	58.7	
		actinR2	CCGACGACGCTCCGCTTCCAACT		
al-3	NCU01427.3	al-3F1	AAAAGGAAATGGAACACG	57.2	
		al-3R1	GGATGCCGAAGATGGAGT		
		al-3F2	CGCCGCGGCTTCCCTGTC	58.3	
		al-3R2	TCACTCCTTCTTCCCATTCTTCT		
cyh-2	NCU03806.3	cyh-2F	CTTCATCGGCACCACCAGCAGACA	61.3	
		cyh-2R	AATGACACCACCGGCCTCCTTGAT		
his-7	NCU07156.3	his-6F1	ATGCCGACCGTTCACCT	59.8	
		his-6R1	TTCGTCGGCGCCTTGTTCGTAGTA		
		his-6F2	ACCGCAACGTCCGCAACC	58.0	
		his-6R2	GATCTCCCTCAAACTGTCTG		
ilv-1	NCU04579.3	ilv-1F1	ATGCTCGCTCCCTCCCTTCTG	61.6	
		ilv-1R1	GATGGCCTCACCGACGCTCAA		
		ilv-1F2	CTTCCCGGTTCCTCCTCCTAC	60.5	
		ilv-1R2	ATCCGCTCTATACCCCTTCTTCAT		
mus-18	NCU08850.3	mus-18F1	ATGCCTTCCCGTAAATC	55.3	
		mus-18R1	TGACTGGCAAAGGGAAACAT		
		mus-18F2	TGGAACGAAAAGTACGGCATCAG	60.3	
		mus-18R2	ACTCCCCTTCATCCTCCACCTCTT		
ro-4	NCU04247.3	ro-4F1-a	GCGGGAGACGACGTACCAAAATG	61.4	
		ro-4R1-a	CGCCGCCCACTCCTTCTCCT		
		ro-4F2-a	GACCTTCAACGTACCTGCTCT	57.0	
		ro-4R2-a	ATGATATCCGGATTCTCGTG		
sod-2	NCU01213.3	sod-2F	CTCGCAAGCTCCTCTCCAG	58.2	
		sod-2R	ACCATAACCGCAGTCATAGCA		
vma-3	NCU01332.3	vma-3F	GTCCCGACTCTTCCCAACAACACG	60.5	
		vma-3R	CGCGAAACCAGCAGCAAGACCA		
LG-VI					
Bml	NCU04054.3	b-tubF1	CGCGTCGGCTTTATCACCAC	60.2	
		b-tubR1	GGGAGGGCACGACGGAGAAG		
		b-tubF2	GTCCGTCGCGAGGCTGAGG	60.7	
		b-tubR2	AACACTGTAACCCGCAACGAAGAA		
1Broad Institute Neurospora crassa database.
2Forward and reverse primers are indicated by capital F and R, respectively. In case several primer pairs have been used for amplification of different parts of the same gene, each primer in the pair is assigned to the same number which follows after capital F or R.
